# Reliability of Lupus Anticoagulant and Anti-phosphatidylserine/prothrombin Autoantibodies in Antiphospholipid Syndrome: A Multicenter Study

**DOI:** 10.3389/fimmu.2019.00376

**Published:** 2019-03-05

**Authors:** Savino Sciascia, Massimo Radin, Irene Cecchi, Elena Rubini, Anna Scotta, Roberta Rolla, Barbara Montaruli, Patrizia Pergolini, Giulio Mengozzi, Emanuela Muccini, Simone Baldovino, Michela Ferro, Antonella Vaccarino, Michael Mahler, Elisa Menegatti, Dario Roccatello

**Affiliations:** ^1^Center of Research of Immunopathology and Rare Diseases- Coordinating Center of Piemonte and Valle d'Aosta Network for Rare Diseases, Department of Clinical and Biological Sciences, University of Turin and S. Giovanni Bosco Hospital, Turin, Italy; ^2^Nephrology and Dialysis Unit, S. Giovanni Bosco Hospital and University of Turin, Turin, Italy; ^3^Department of Clinical and Biological Sciences, School of Specialization of Clinical Pathology, University of Turin, Turin, Italy; ^4^AOU Clinical Chemistry Laboratory, “Maggiore della Carità” University Hospital, Amedeo Avogadro University of Eastern Piedmont, Novara, Italy; ^5^AO Ordine Mauriziano, Turin, Italy; ^6^Department of Clinical Biochemistry, AOU Cittá della Salute e della Scienza, Turin, Italy; ^7^Hematology Division, and S. Giovanni Bosco Hospital, Turin, Italy; ^8^Inova Diagnostics, Research and Development, San Diego, CA, United States

**Keywords:** antiphosphospholipid syndrome, Antiphospholipid Antibodies, Lupus Anticoagulant, aPS/PT, thrombosis, laboratory, diagnostic performance, reliability

## Abstract

**Background:** Is it well-known that one of the major drawbacks of Lupus Anticoagulant (LA) test is their sensitivity to anticoagulant therapy, due to the coagulation based principle. In this study we aimed to assess the reproducibility of LA testing and to evaluate the performance of solid assay phosphatidylserine/prothrombin (aPS/PT) antibodies.

**Methods:** We included 60 patients that fulfilled the following inclusion criteria: (I) diagnosis of thrombotic antiphospholipid syndrome (APS); (II) patients with thrombosis and (a) inconstant previous LA positivity and/or (b) positivity for antiphospholipid antibodies (aPL) at low-medium titers [defined as levels of anti-β2Glycoprotein-I or anticardiolipin (IgG/IgM) 10–30 GPL/MPL] with no previous evidence of LA positivity. aPL testing was performed blindly in 4 centers undertaking periodic external quality assessment.

**Results:** The 60 patients enrolled were distributed as follows: 43 (71.7%) with thrombotic APS, 7 (11.7%) with thrombosis and inconstant LA positivity and 10 (16.7%) with low-medium aPL titers. Categorical agreement for LA among the centers ranged from 0.41 to 0.60 (*Cohen's kappa* coefficient; moderate agreement). The correlation determined at the 4 sites for aPS/PT was strong, both quantitatively (Spearman rho 0.84) and when dichotomized (*Cohen's kappa* coefficients = 0.81 to 1.0). Discordant (as defined by lack of agreement in ≥3 laboratories) or inconclusive LA results were observed in 27/60 (45%) cases; when limiting the analysis to those receiving vitamin K antagonist (VKA), the level of discordant LA results was as high as 75%(15/20). Conversely, aPS/PT testing showed an overall agreement of 83% (up to 90% in patients receiving VKA), providing an overall increase in test reproducibility of +28% when compared to LA, becoming even more evident (+65%) when analyzing patients on VKA. In patients treated with VKA, we observed a good correlation for aPS/PT IgG testing (*Cohen's kappa* coefficients = 0.81–1; Spearman rho 0.86).

**Conclusion:** Despite the progress in the standardization of aPL testing, we observed up to 45% of overall discrepant results for LA, even higher in patients on VKA. The introduction of aPS/PT testing might represent a further diagnostic tool, especially when LA testing is not available or the results are uncertain.

## Introduction

Since clinical features of Antiphospholipid Syndrome (APS) (thrombosis and pregnancy complications) are common in the general population and often related to other underlying factors, the diagnosis of APS requires next to clinical assessment the detection of persistently positive Antiphospholipid Antibodies (aPL). Thus, reliable laboratory tests with good clinical and analytical performance reproducibility are required. There is a large variety of assays available to assess aPL, but despite progress, standardization is still not optimal (1–3).

Lupus anticoagulant (LA) has been shown to be the strongest risk factor for aPL-related clinical manifestations (4), and the correct interpretation of this functional assay is crucial for diagnosis of APS. However, testing patients during treatment with vitamin K antagonists (VKA) or other oral anticoagulants remains a contentious issue and has been discouraged by official guidelines (5–7) because of interpretational problems affecting the mixing test. Besides, the clinical significance of low aPL titer and/or weak LA positivity, especially when detected in patients receiving anticoagulation [either VKA or direct anticoagulant agents (DOAC)], remains uncertain and certainly needs a more thorough evaluation.

More recently, the family of aPL has been expanded to include a heterogeneous group of autoantibodies whose specificity is directed to proteins involved in coagulation or to a complex of these proteins with phospholipids (8). Among others, autoantibodies that recognize a phosphatidylserine/prothrombin (aPS/PT) complex have been reported to be associated with APS and may have diagnostic relevance in these settings (9, 10). However, since aPS/PT antibodies are not currently included in the current APS classification criteria (11), aPS/PT antibodies are not assessed in all patients suspected to suffer from APS. Given the importance of aPL confirmation to improve the interpretability of laboratory test results for clinical trials and research studies, the objective of this study was to assess the reproducibility of LA and aPS/PT antibody testing when performed in different expert centers and to assess the diagnostic performance of these tests in different clinical settings of APS.

## Methods

### Patients

We chart-reviewed patients with thrombotic events who tested persistently positive for at least one aPL (more than two occasions over a time of more than 12 weeks) that presented at San Giovanni Bosco Hospital in the last 5 years. The study was performed in compliance with the Declaration of Helsinki; approval from the ethic committee was not required according to the local and national guidelines. We enrolled 60 patients who met one of the following inclusion criteria:

1. Fulfilled the diagnosis of thrombotic APS defined as per Sydney criteria (11).
2. Patients with thrombosis and suspected APS not completely fulfilling the laboratory criteria (11), as follows: (a) inconsistent previous LA positivity; and/or (b) low-medium aPL titers [defined as levels of anticardiolipin (aCL) IgG/IgM or anti-β2-glycoprotein I (aβ2GPI) IgG/IgM antibodies 10–30 GPL/MPL]. Clinical and laboratory characteristics were retrospectively collected.

### Previous Autoantibody Detection

The aPL profile, at the diagnosis, included aCL, LA, and aβ2GPI antibodies.

The aCL and aß2GPI (IgG and IgM) were detected by commercial ELISA (Inova Diagnostics, Inc., San Diego, CA, US). Plasma samples were tested for the presence of LA according to the recommended criteria from the International Society on Thrombosis and Haemostasis (ISTH) Subcommittee on Lupus Anticoagulant/Phospholipid-Dependent Antibodies (12, 13).

### Study Design

LA and aPS/PT testing was performed in a blind fashion in four centers of the “Antiphospholipid Antibodies Regional Consortium” in northwest Italy: San Giovanni Bosco Hospital, Turin, Italy, A.O.U. Città della Salute e della Scienza, Turin, Italy, A.O. Ordine Mauriziano, Turin, Italy, and A.O.U. Maggiore della Carità, Novara, Italy (14).

LA was tested with the detection of two different reagents, used as screening and confirmatory tests, Silica Clotting Time HemosIL and dRVVT Screen and Confirm HemosIL, respectively (Instrumentation Laboratory, Bedford, MA, USA). Both tests were automated on ACL TOP 750 LAS instruments and results were normalized by means of plasma pools obtained from healthy donors without any deficit in coagulation factors, as per the current criteria from the ISTH Subcommittee on LA-Phospholipid-dependent antibodies (12, 13).

Both IgG and IgM aPS/PT were assayed using commercial ELISA kits (QUANTA Lite®, Inova Diagnostic), in accordance with manufacturer's instructions. Samples were considered positive for aPS/PT IgG/IgM if tested >30 U.

Agreement was defined when all four laboratories had a concordant binomial result (positive/negative), both for LA and aPS/PT IgG/IgM testing.

### Statistical Analysis

Categorical variables are presented as number (%) and continuous variables are presented as mean (S.D.). Categorical agreement and degree of linear association was analyzed. The significance of baseline differences was determined by the chi-squared test, Fisher's exact test or the unpaired *t*-test, as appropriate. A two-sided *p*-value < 0.05 was statistically significant. All statistical analyses were performed using SPSS version 19.0 (IBM, Armonk, NY, USA).

## Results

Demographic, clinical and laboratory characteristics of the 60 patients enrolled in the study are summarized in Table 1.

**Table 1:** Characteristics of the patients included in the study.

	**APS patients (43; 72%)**	**Suspected APS (17; 28%)**
**ANAGRAPHIC**		
Mean age (±S.D.) at data collection	45.7 (±11.9)	51.9 (±7.3)
Females	30 (69.8%)	11 (64.7%)
**CLINICAL MANIFESTATIONS**		
Arterial thrombosis	21 (48.8%)	5 (29.4%)
Venous thrombosis	26 (60.5%)	12 (70.6%)
**aPL PROFILE AT DIAGNOSIS**		
LA (positive, *n*)^*^	37 (86%)	11 (64.7%)
aCL (IgG/M)^*^	22 (51.2%)	7 (41.2%)
aβ2GPI (IgG/M)^*^	23(53.5%)	6 (35.3%)
**ANTICOAGULANT THERAPY AT THE MOMENT OF TESTING**		
VKA (warfarin)	18 (41.9%)	2 (11.8%)
LMWH	8 (18.6%)	2 (11.8%)
DOAC	13 (30.2%)	0
Anti-platelets therapy	17 (39.5%)	13 (76.5%)

*SD, Standard Deviation; APS, Antiphospholipid Syndrome; aPL, Antiphospholipid Antibodies; LA, Lupus Anticoagulant; aCL, Anticardiolipin Antibodies; anti-β2GPI, Anti- β2Glycoprotein I antibodies; VKA, Vitamin K antagonists; LMWH, Low Molecular Weight Heparins; DOAC, Direct Anticoagulants*.

* *When considering patients with suspected APS: defined as inconsistent LA positivity and/or low levels of ACA IgG/IgM or anti-β2GPI IgG/IgM antibodies 10–30 GPL/MPL*.

Briefly, mean age at data collection was 49.9 years old (SD ± 10.9) (females: males = 71.7%: 28.3%). Forty-three patients (71.7%) had a confirmed diagnosis of thrombotic APS (arterial 58.1%; venous 56.3%), and 17 patients presented with thrombosis and inconsistent LA positivity [7/17 (41.2%)] and/or with low-medium titers [10/17(58.8%)]. In the latest, 10/17 patients with suspected APS were tested positive (titer > 30 UI) for aPS/PT, IgG and/or IgM.

Overall, categorical agreement for LA among all the four centers, as expressed by Cohen's *kappa* coefficients, ranged from 0.41 to 0.60 (corresponding to moderate agreement). The correlation among quantitative results for aPS/PT IgG/IgM was strong (Spearman *rho* 0.84; when dichotomizing for positive vs. negative results, Cohen's *kappa* coefficients = 0.81–1.00).

**Figure 1:**
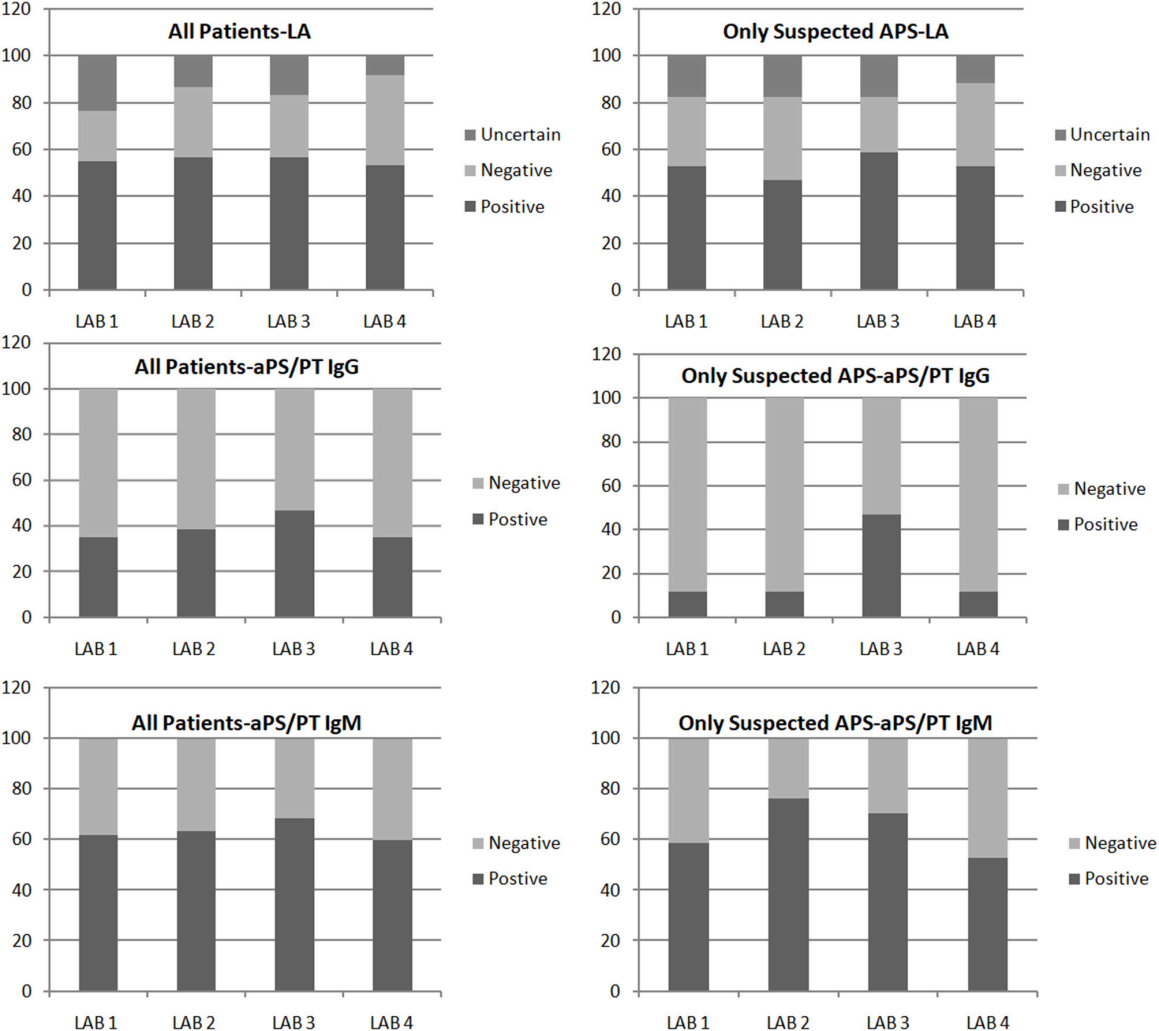
Results of lupus anticoagulant (LA) and anti-PS/PT antibody obtained in four laboratories. Results are summarized for all the patients included in the study **(Left)** and for patients with suspected APS **(Right)**.

Overall categorical agreement is resumed in Figure 1.

We observed 27 (45.0% of the total) cases (15/20, 75% patients on VKA) in which LA results were discordant (defined by lack of agreement) or inconclusive. Conversely, in those cases, we observed a good correlation for aPS/PT IgG/IgM testing (Cohen's *kappa* coefficients = 0.81–1.00, Spearman *rho* 0.86).

When considering previous LA testing, we observed a statistically significant higher agreement among centers of LA testing if LA testing was previously positive [LA previously positive testing vs. negative: full agreement among centers 74.5% vs. 30.7% (*chi Square* test *p* < 0.05)]. Interestingly, the level of agreement of aPS/PT IgG/IgM among centers was similar regardless of previous LA testing [LA previously positive vs. negative: full agreement among centers 85.1% vs. 92.3% (*chi Square* test *p* = 0.49)].

When stratifying patients according to the inclusion criteria, we observed that in patients with confirmed diagnosis of APS, LA, and aPS/PT (IgG/IgM) agreements were 24/43 (55.8%) and 40/43 (93.0%), respectively. Conversely, in patients with thrombosis not completely fulfilling the Sydney laboratory criteria, we found aPL testing agreement among the four centers as follows: LA 9/17 (52.9%) and aPS/PT IgG/IgM 11/17 (64.7%).

## Discussion

The diagnosis and consequent management as well as the classification of APS relies on the identification of persistent aPL positivity in patients with thrombosis and/or pregnancy morbidity (11). Among aPL tests, LA has been shown to be the strongest risk factor for thrombotic events (15) and LA testing should always be performed in parallel with aCL and aβ2GPI (3, 16–18) when a patients is investigated for APS.

However, despite significant progress in LA testing thanks to the updated guidelines of the ISTH (12, 13), LA testing still suffers from some shortcomings and remain much more labor intensive and complicated to perform compared to immunoassays.

In our study, when testing for LA in a blind fashion in four centers all undergoing regular external quality assessment (EQA) (14), we observed that up to 45% of LA positive samples were not unanimously identified. When limiting the analysis to patients with VKA, the observed level of agreement dropped to 55%.

Is it well-known that one of the major drawbacks of LA tests is their sensitivity to anticoagulant therapy (such as VKA, and DOAC), due to the coagulation based principle. Preferably, tests should be postponed until therapy is stopped; however, in the real world, requests during therapy still occur very frequently with potentially false-positive or false-negative results (13, 19). In addition, it might be logistical inconvenient for the patients to switch (or stop) anticoagulant therapy for LA testing purpose.

When a thrombotic event occurs in patients suspected for APS with inconsistent LA positivity and/or with low-medium aPL titers, clinical management can be challenging, as no consensus exists on the choice and, more critically, the duration of anticoagulation in this setting. In this study, when analyzing patients not completely fulfilling the criteria for APS, we observed a level of LA agreement of only 53%, supporting the need of further diagnostic tool to help physicians in the management of these patients.

Autoantibodies directed toward PS/PT complexes have been extensively studied for their diagnostic and prognostic utility in patients with suspected APS (9). Due to the observation that anti-prothrombin antibodies associate significantly with LA (20–24), several studies have sought to define the diagnostic relevance of these antibodies in APS (9, 23, 25, 26). Recent evidence support that while aPS/PT are frequently found in patients with LA, their association with thrombosis seems to be independent of the presence of LA (27).

Among the so-called extra-criteria aPL tests, besides aPS/PT, antiβ2GPI-domain1 antibodies have been also proposed to potentially improve the diagnostic accuracy in patients with suspected APS (28, 29), especially when assessing the risk for both thrombosis and pregnancy morbidity. Other antibody specificities, such as anti-annexin A5 and anti-vimentin antibodies, might be considered for thrombotic risk assessment only in selected patients, particularly when other aPL tests are negative and in the presence of clinical signs and/or symptoms strongly suggestive for APS (26, 30).

In our cohort, aPS/PT testing showed an overall agreement of 83% (up to 90% in patients receiving VKA), providing an overall increase in test reproducibility of +28% when compared to LA, becoming even more evident (+65%) when analyzing patients on VKA. These observations have important implications. On the one hand, LA testing remains a cornerstone for APS diagnosis. On the other, ongoing efforts to reduce the LA testing interlaboratory/interassay variations remain important. Taking into account the methodological shortcomings of LA, aPS/PT might represent a reliable and reproducible test, even during VKA or when APS diagnosis in uncertain. Besides the diagnosis, these findings also might have significant implications for classification criteria and therefore for clinical trials of new treatments.

Besides, albeit investigating the impact of aPS/PT testing on the management of patients with suspected APS was out of the scope of this study, one might note that up to nearly 60% of the patients with suspected APS were found positive for aPS/PT. From a speculative point of view, this observation might support a role for aPS/PT testing when APS is suspected but currently classification criteria aPL are not fully informative/reliable.

Although our investigation suffers for some limitations (cross-sectional approach limiting the analysis of the longitudinal fluctuation in aPL positivity; limited sample size; no further analysis on the level of agreement for aCL and aβ2GPI), the strengths of this study relies on the blind approach of aPL testing, performed in four different centers all undergoing periodic EQA. Besides, in this investigation, we evaluated the robustness of aPS/PT ELISA testing in different clinical settings, including patients suspected for APS but tested negative/low-titers for aCL and aβ2GPI antibodies. In such cases, a further diagnostic tool for APS with reliable performances might be crucial to guide the diagnostic process and to avoid under/over treatment (31). Finally, testing for aPS/PT by a commercial kit was proven to be a reproducible and accurate test for the detection of aPS/PT, bringing the added advantage of shorter running times when compared to in-house assays (32).

In conclusion, despite the progress in the standardization of aPL testing, we observed up to 45% of overall discrepant results for LA, even higher in patients on VKA. Our findings showed that the persistence of significant discordance in the reliability of LA testing. The introduction of aPS/PT antibodies in the diagnostic process of APS might represent a further valuable diagnostic tool, especially when LA is not available or reliable. In addition, detection of aPS/PT antibodies provides another tool which can complement and support current testing with aCL and aβ2GPI assays, and further help guiding clinical management.

## Data Availability

This author takes responsibility for all aspects of the reliability and freedom from bias of the data presented and their discussed interpretation.

## Ethics Statement

This study was carried out in accordance with the recommendations for rare diseases in Piedmont Region, Northwest Italy with written informed consent from all subjects. All subjects gave written informed consent in accordance with the Declaration of Helsinki.

## Author Contributions

SS, MR, IC, ER, MM, and DR drafted the manuscript, figures, and tables and critically reviewed the manuscript. AS, RR, BM, PP, GM, EMu, SB, MF, AV, and EMe participated in laboratory testing and critically reviewed the manuscript.

### Conflict of Interest Statement

The authors declare that the research was conducted in the absence of any commercial or financial relationships that could be construed as a potential conflict of interest.
